# Biochemical Mechanisms and Microorganisms Involved in Anaerobic Testosterone Metabolism in Estuarine Sediments

**DOI:** 10.3389/fmicb.2017.01520

**Published:** 2017-08-11

**Authors:** Chao-Jen Shih, Yi-Lung Chen, Chia-Hsiang Wang, Sean T.-S. Wei, I-Ting Lin, Wael A. Ismail, Yin-Ru Chiang

**Affiliations:** ^1^Biodiversity Research Center, Academia Sinica Taipei, Taiwan; ^2^Bioresource Collection and Research Center, Food Industry Research and Development Institute Hsinchu, Taiwan; ^3^Environmental Biotechnology Program, Department of Life Sciences, College of Graduate Studies, Arabian Gulf University Manama, Bahrain

**Keywords:** androgen, biodegradation, estrogen, estuary, Illumina MiSeq, sediment, testosterone, *Thauera*

## Abstract

Current knowledge on the biochemical mechanisms underlying microbial steroid metabolism in anaerobic ecosystems is extremely limited. Sulfate, nitrate, and iron [Fe (III)] are common electron acceptors for anaerobes in estuarine sediments. Here, we investigated anaerobic testosterone metabolism in anaerobic sediments collected from the estuary of Tamsui River, Taiwan. The anaerobic sediment samples were spiked with testosterone (1 mM) and individual electron acceptors (10 mM), including nitrate, Fe^3+^, and sulfate. The analysis of androgen metabolites indicated that testosterone biodegradation under denitrifying conditions proceeds through the 2,3-*seco* pathway, whereas testosterone biodegradation under iron-reducing conditions may proceed through an unidentified alternative pathway. Metagenomic analysis and PCR-based functional assays suggested that *Thauera* spp. were the major testosterone degraders in estuarine sediment samples incubated with testosterone and nitrate. *Thauera* sp. strain GDN1, a testosterone-degrading betaproteobacterium, was isolated from the denitrifying sediment sample. This strain tolerates a broad range of salinity (0–30 ppt). Although testosterone biodegradation did not occur under sulfate-reducing conditions, we observed the anaerobic biotransformation of testosterone to estrogens in some testosterone-spiked sediment samples. This is unprecedented since biotransformation of androgens to estrogens is known to occur only under oxic conditions. Our metagenomic analysis suggested that *Clostridium* spp. might play a role in this anaerobic biotransformation. These results expand our understanding of microbial metabolism of steroids under strictly anoxic conditions.

## Introduction

Steroids, a class of triterpenoids produced mainly by eukaryotes, are ubiquitous and abundant in nature. Steroids include sterols, steroid hormones, and bile acids. These compounds are characterized by a planar and relatively rigid carbon skeleton composed of four fused alicyclic rings. Steroid hormones [e.g., estrone (E1), testosterone, and progesterone] produced by humans and livestock are discharged into aquatic environments through various routes, including wastewater treatment plant effluent and runoff from manure applications (Lorenzen et al., 2004). Among these steroid hormones, androgens typically occur in effluents from wastewater treatment plants and in rivers worldwide, at concentrations ranging from nanograms to micrograms per liter (Yamamoto et al., 2006; Chang et al., 2009, 2011; Fan et al., 2011). The occurrence of steroid hormones in aquatic ecosystems is associated with environmental concerns because of their adverse effects on animal physiology and behavior, even at extremely low concentrations [e.g., sub-nanograms per liter for 17β-estradiol (E2) (Massart et al., 2006; Ghayee and Auchus, 2007) and sub-micrograms per liter for testosterone (Koger et al., 2000)]. Several studies have reported that androgen exposure caused masculinization of aquatic wildlife (Howell et al., 1980; Bortone et al., 1989; Parks et al., 2001; Orlando et al., 2004). Moreover, androgens elicit both odorant and pheromonal responses in fish at extremely low concentrations (Adams et al., 1987; Sorensen and Stacey, 1999). For instance, precocious male Atlantic salmon exhibit an odorant response to testosterone at concentrations as low as 0.003 ng/L (Moore and Scott, 1991).

Estuarine and coastal environments near large cities worldwide receive various pollutants, including steroid hormones, through sewage effluent and industrial wastewater (Ying and Kookana, 2003). The persistence and metabolic fate of steroid hormones in these environments is critical because estuarine and coastal ecosystems provide habitats for numerous organisms and support high productivity. Both physical adsorption by sediment particles and microbial biodegradation are crucial for steroid hormone elimination from aquatic ecosystems (Czajka and Londry, 2006). Steroids degrade more gradually in anaerobic sediments than in aerobic sediments (Ying and Kookana, 2003). River and marine sediments, particularly anaerobic sediments, are thus considered potential reservoirs for these hydrophobic and recalcitrant compounds (Peck et al., 2004). Similarly, steroids are persistent in anaerobic soils (Colucci et al., 2001). Consequently, it is important to investigate and expand scientific knowledge regarding biodegradation of steroids in aerobic and anaerobic ecosystems; expanded knowledge can enable the development of bioremediation technologies for polluted environments.

**FIGURE 1:**
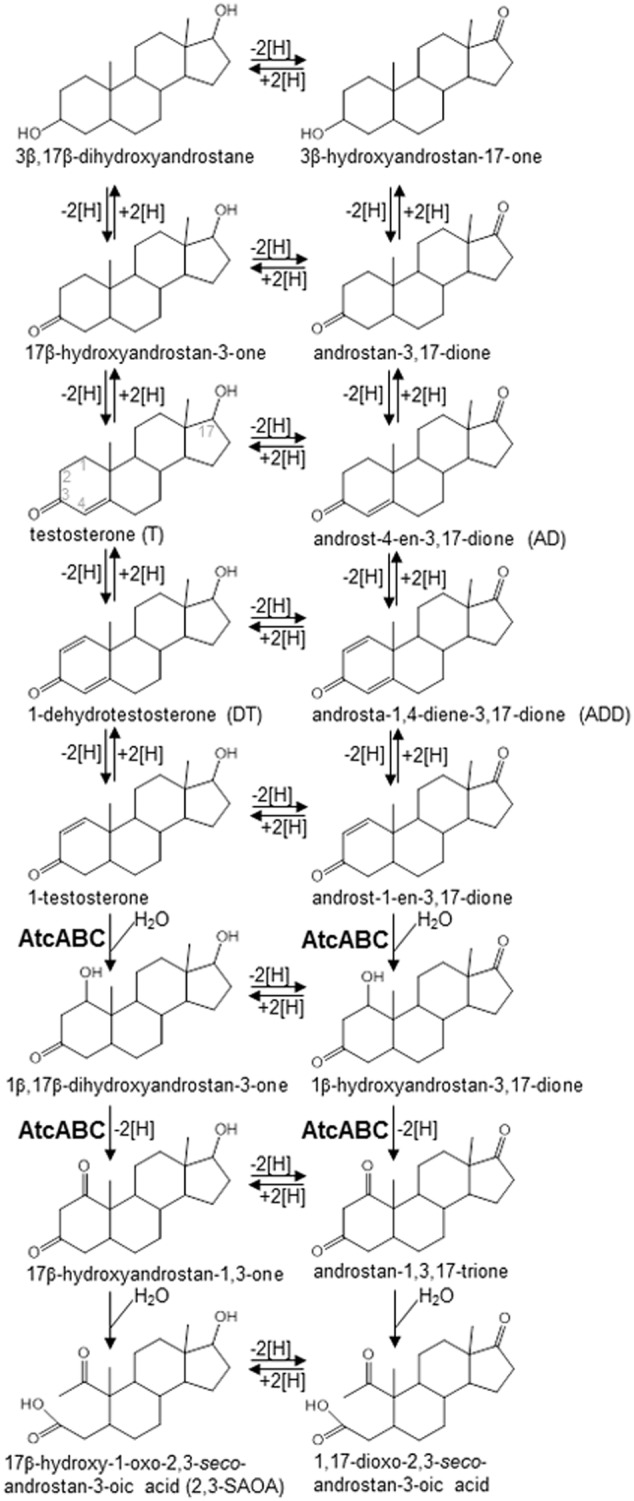
Androgen metabolites identified in *Steroidobacter denitrificans* DSM 18526 grown anaerobically on testosterone. AtcABC, 1-testosterone hydratase/dehydrogenase. The carbon numbering system of steroids is as shown for testosterone.

Although the biochemical mechanisms and microorganisms involved in aerobic androgen degradation in the environment have been extensively studied (Horinouchi et al., 2012; Bergstrand et al., 2016; Chen et al., 2016), anaerobic androgen biodegradation remains unclear (Yang et al., 2016). All androgen-degrading anaerobes isolated thus far are denitrifiers, including *Steroidobacter* (*Sdo.*) *denitrificans* (Fahrbach et al., 2010), *Sterolibacterium* (*Stl.*) *denitrificans* (Wang et al., 2014), and *Thauera terpenica* (Foss and Harder, 1998). These bacteria degrade androgens through the oxygenase-independent 2,3-*seco* pathway (Yang et al., 2016). Various androgen metabolites (Chiang et al., 2010; Fahrbach et al., 2010; Leu et al., 2011; Wang et al., 2013) (see **Figure 1** for their structures) have been identified in *Sdo. denitrificans* DSM 18526, a model microorganism for anaerobic testosterone catabolism. Although the redox reactions at C-1/C-2, C-4/C-5, 3-oxo, and 17-hydroxyl groups occur commonly in both the anaerobic (2,3-*seco* pathway) and aerobic (9,10-*seco* pathway) testosterone catabolic pathways (Ismail and Chiang, 2011; Horinouchi et al., 2012), many of the intermediates [e.g., 1β,17β-dihydroxyandrostan-3-one, 17β-hydroxyandrostan-1,3-dione, 17β-hydroxy-1-oxo-2,3-*seco*-androstan-3-oic acid (2,3-SAOA), and their 17-oxo structures] identified in the anaerobically testosterone-grown *Sdo. denitrificans* are characteristic to the anaerobic 2,3-*seco* pathway and do not occur in the aerobic 9,10-*seco* pathway. These characteristic compounds can be exploited as signature metabolites for studying anaerobic testosterone degradation in the environment. Some catabolic genes associated with anaerobic androgen catabolism have been identified in the *Sdo. denitrificans* genome (Yang et al., 2016). Furthermore, a bifunctional molybdoenzyme, 1-testosterone hydratase/dehydrogenase (AtcABC), catalyzing the hydration reaction at C-1/C-2 of 1-testosterone and the subsequent dehydrogenation reaction was purified from *Sdo. denitrificans* (Yang et al., 2016). The phylogenetic analysis of the sequences of AtcABC suggested that this enzyme belongs to the xanthine oxidase family containing molybdopterin, FAD, and iron-sulfur clusters. The corresponding genes (*atcA, atcB*, and *atcC*) are clustered in the *Sdo. denitrificans* genome. In addition to *Sdo. denitrificans, atcABC*-like genes are present in the genomes of various androgen-degrading denitrifiers such as *Stl. denitrificans* and *T. terpenica*. This enabled the design of degenerate primers specific to *atcA* (Yang et al., 2016).

Anaerobes may use various electron acceptors to degrade hydrocarbons, depending on their environments. Nitrate (micromolar range) (Fan et al., 2006), iron (micromolar range) (Boyle et al., 1977), and sulfate (millimolar range) (Fan et al., 2012) are abundant in estuarine ecosystems. Whether testosterone is biodegradable in estuarine sediments under iron- and sulfate-reducing conditions remains unclear. In this study, we determined whether the microbial populations in estuarine sediments collected from the Tamsui River, Northern Taiwan, have anaerobic testosterone degradation capacity. To enrich testosterone-degrading microbial populations, the estuarine sediment samples were incubated with 1 mM testosterone as well as different electron acceptors, including nitrate, Fe^3+^, and sulfate. To investigate the biochemical mechanisms and microorganisms involved in anaerobic testosterone metabolism in the estuarine sediments, we used the following approaches: (i) identification of androgen metabolites through ultra-performance liquid chromatography–electrospray ionization–mass spectrometry (UPLC–ESI–MS) and high-performance liquid chromatography with an ultraviolet detector (HPLC–UV), (ii) phylogenetic identification of testosterone-degrading bacteria through Illumina Miseq sequencing, and (iii) detection of the characteristic catabolic gene *atcA* through PCR.

## Materials and Methods

### Sampling Site and Sample Collection

Approximately six million people reside in the basin of the Tamsui River, Taipei, Taiwan. The estuary of the Tamsui River receives sewage discharges and waste effluent from the Taipei metropolitan area. The effluent of the Dihua Sewage Treatment Plant, containing approximately 0.9 nM androgens, including testosterone and androst-4-en-3,17-dione (AD) (Yang et al., 2016), is discharged into the Tamsui River. Our sampling site, Guandu (25°6′59.56″N, 121°27′46.99″E), with salinity of 5–22 parts per thousand (ppt) (Fan et al., 2006; Hsieh et al., 2010), is located in the upper estuary where the Keelung River meets the main channel of the Tamsui River. Sewage discharges from upstream and seawater intrusion mix here. The chemical parameters and microorganisms present in Guandu have been reported (Wu, 1999; Fan et al., 2006, 2012; Kao et al., 2013). The vertical profiles of environmental factors (e.g., nitrate and nitrite concentrations) as well as denitrifying bacteria in the Guandu sediments were investigated by Fan et al. (2006), and the data indicated that nitrate and denitrifying bacteria were largely distributed in the subsurface layer. Fan et al. (2012) also demonstrated that reduced sulfur compounds began to accumulate and the abundance of sulfur-reducing bacteria began to increase at 10 cm below the sediment surface. In the current study, four sediment cores were collected from Guandu. The sediment samples were collected using polyvinyl chloride corers (7.5-cm diameter). During the low tide on July 20, 2015, the corers were pressed down approximately 50 cm into the sediments and sealed with a rubber stopper immediately after collection. River water samples (20 L) were collected from Guandu on the same day. Sterilized 10-L glass bottles were filled with the river water and tightly capped to avoid headspace formation. The sediment and river water samples were carried to the laboratory within 1 h of collection and processed immediately.

### Incubation of Anaerobic Sediments with Testosterone

The anaerobic microcosms were prepared in an anaerobic chamber containing 95% (v/v) nitrogen gas and 5% hydrogen gas. Each sediment core was cut into three sections: subsurface layer (0–5 cm depth), middle layer (6–10 cm depth), and bottom layer (11–15 cm depth). Vertical sectioning of the sediment cores was based on the vertical distributions of chemicals and anaerobic bacteria in the Guandu sediments (Fan et al., 2006, 2012). Sediment (100 g) was added to the 1-L glass bottles containing river water (900 mL), and the bottles were sealed with butyl rubber stoppers; the gas phase was then exchanged to N_2_:CO_2_ (80:20). The androgen content in the original sediment–river water mixtures, not spiked with testosterone or electron acceptors, was determined using LC–MS, as described by Yang et al. (2016), whereas nitrate, Fe^3+^, and sulfate content in the mixtures was determined as described in the subsequent sections. The chemical properties in the original sediment–river water mixtures are shown in Supplementary Table S1. The mixed sediment–river water samples (1 L) were incubated under the following conditions: sediment–river water mixture (0–5 cm sediment depth) with testosterone (1 mM) or nitrate (NaNO_3_, 10 mM) or both; sediment–river water mixture (6–10 cm sediment depth) with testosterone (1 mM) or ferric pyrophosphate (Fe_4_O_21_P_6_, 2.5 mM; ferric ion = 10 mM) or both; and sediment–river water mixture (11–15 cm sediment depth) with testosterone (1 mM) or sulfate (Na_2_SO_4_, 10 mM) or both. In the testosterone-spiked treatments, the bottles containing testosterone (288 mg) were autoclaved before the addition of the sediment–river water mixture. The stock solutions (1 M) of individual electron acceptors were prepared anaerobically and autoclaved before use. The abiotic controls were prepared by anaerobic incubation of the autoclaved sediment–river water mixtures with testosterone (1 mM) and individual electron acceptors (10 mM). All microcosms were reduced with 1 mM Na_2_S immediately before anaerobic incubation in dark at 25°C with stirring at 160 rpm for 5 weeks. The sediment–river water mixtures (10 mL) were sampled from the bottles every 2 days and stored at -80°C before use. The determination of residual nitrate, Fe^3+^, sulfate, and androgen metabolites in the samples are described in the subsequent sections. The bacterial 16S rRNA and functional *atcA* genes in the sediment–river water samples were analyzed through Illumina MiSeq sequencing and PCR-based functional assays, respectively.

### Chemical Characteristics of Sediment–River Water Mixtures

The samples (3 mL) of the sediment–river water mixtures were centrifuged at 10,000 rpm for 15 min, and the supernatant was filtered through a 0.45-μm Minisart Syringe filter (Sartorius Stedim). Salinity was determined using a refractometer (SA28T; Rocker Scientific). Nitrate content in the samples was determined using a cadmium reduction method, according to manufacturer’s instructions (Nitrate Reagent Kit HI93728-01; Hanna Instruments). Fe^3+^ concentration in the samples was determined using a photometric method, according to manufacturer’s instructions (Spectroquant Iron Test 100796; Merck). Sulfate content in the samples was measured using a photometric method, according to manufacturer’s instructions (Spectroquant Sulfate Test 101812; Merck).

### UPLC–ESI–MS Analysis of Androgenic Metabolite Profiles

Samples (1 mL) retrieved from the sediment–river water treatments were extracted three times with equal volumes of ethyl acetate. The extracts were pooled, the solvent was evaporated, and the residues were re-dissolved in 100 μL of methanol. The ethyl acetate extractable samples were analyzed through UPLC–ESI–MS. MS data were collected in the +ESI mode in separate runs on a Waters Synapt G2 HDMS mass spectrometer operated in scan mode from 50 to 600 *m*/*z*. Separation was achieved on a reversed-phase C_18_ column (Acquity UPLC BEH C18, 1.7 μm, 100 × 2.1 mm; Waters), with a flow rate of 0.4 mL/min at 35°C (column oven temperature). The mobile phase comprised a mixture of solvents A [2% (v/v) acetonitrile containing 0.1% formic acid] and B [100% (v/v) methanol containing 0.1% formic acid]. Separation was achieved with a linear gradient of solvent B from 10 to 99% in 8 min, followed by an isocratic step at 99% solvent B for the next 1 min. For ESI–MS, the capillary voltage was set at 3000 V; source and desolvation temperatures at 80°C and 350°C, respectively; and cone and desolvation gas flow rates at 20 and 700 L/h, respectively. The predicted elemental composition of individual intermediates was calculated using MassLynx (Waters).

### HPLC Quantification of Androgen Metabolites

Steroid metabolites in 1 mM testosterone-spiked sediment–river water samples were quantified through HPLC–UV. 17β-Ethinylestradiol (final concentration, 50 μM) was added to sediment–river water samples (1 mL) as an internal control. The separation was achieved on an analytical RP-C_18_ column [Luna PFP(2), 5 μm, 250 × 4.6 mm; Phenomenex] with a flow rate of 0.8 mL/min at 35°C. The mobile phase comprised a mixture of solvents A [100% (v/v) double distilled water] and B [100% (v/v) acetonitrile]. The column was pre-equilibrated with 5% solvent B. The separation was performed with a linear gradient of solvent B from 5 to 60% over 5 min (0–5 min), followed by an isocratic step at 60% solvent B for 8 min (5–13 min), then another linear gradient of solvent B from 60 to 100% over 2 min (13–15 min), and finally an isocratic step at 100% solvent B for 3 min (15–18 min). The steroids were detected in the range of 200–400 nm by using a photodiode array detector. The androgens and estrogens were quantified at 240 and 280 nm, respectively. The quantity of steroids was calculated from their respective peak areas using a standard curve of individual standards. The *R*^2^ values for the standard curves were >0.98.

### Illumina MiSeq Sequencing of Bacterial 16S rRNA Amplicons

DNA was extracted from the frozen sediment–river water samples (5 mL) using a Powersoil DNA isolation kit (MO BIO Laboratories). A 16S amplicon library was prepared according to the Illumina 16S Metagenomic Sequencing Library Preparation Guide^1^ with minor modifications. Genomic sections flanking the V3–V4 region of the bacterial 16S rRNA gene were amplified from 45 sewage treatment samples by using HiFi HotStart ReadyMix (KAPA Biosystems) through PCR (95°C for 3 min; 25 cycles: 95°C for 30 s, 55°C for 30 s, 72°C for 30 s, and 72°C for 5 min). A primer pair flanked by the Illumina Nextera linker sequence was used (forward: 5′-TCGTCGGCAGCGTCAGATGTGTATAAGAGACAGCCTACGGGNGGCWGCAG-3′; reverse: 5′-GTCTCGTGGGCTCGGAGATGTGTATAAGAGACAGGACTACHVGGGTATCTAATCC-3′) (Klindworth et al., 2013). PCR reaction mixtures (50 μL) contained the KAPA HiFi HotStart ReadyMix (2X), 10 pmol of each primer, and 30 ng of template DNA. The PCR products were first separated on an agarose gel (2%, w/v), and those with the expected size (∼445 bp) were excised from the gel and purified using GenepHlow Gel/PCR kit (Geneaid). Illumina Nextera XT Index (Illumina) sequencing adapters were then integrated to the ends of the amplicons through PCR (95°C for 3 min; 8 cycles: 95°C for 30 s, 55°C for 30 s, 72°C for 30 s; and 72°C for 5 min). The final libraries were purified using AMPure XP beads (Beckman Coulter) and quantified using a Qubit dsDNA HS Assay Kit (Life Technologies). The library profiles were then randomly analyzed using an Agilent High Sensitivity DNA Kit on BioAnalyzer. To ensure consistency in pooling, all 45 libraries were subjected to quantitative PCR normalization using KAPA Library Quantification Kits to derive the molar concentrations, and the final library mixture was verified through quantitative PCR. The library pool was sequenced on an Illumina MiSeq V2 sequencer by using a MiSeq Reagent Kit V3 for paired-end reads (2 × 300 bp). For each sediment treatment, an average of 327 361 reads was obtained. We analyzed the sequencing data in Illumina BaseSpace cloud service using the BaseSpace app 16S Metagenomics (version 1.01, Illumina) (Illumina, 2014). The reads were classified against the Illumina-curated version of May 2013 Greengenes taxonomy database by using the Ribosomal Database Project (RDP) naïve Bayesian algorithm^2^. The nucleotide sequence dataset was deposited in the NCBI Sequence Read Archive under the accession number PRJNA382280.

### Detection of the *atcA* Genes Using PCR

*atcA* gene fragments were amplified with degenerate primers (forward: 5′-GGCASCGYYSAGTTCATCGACAA-3′; reverse: 5′-GCCGCTGTCRTAYTCRTTSCCGCTSGG-3′) by using PCR (94°C for 5 min; 35 cycles: 94°C for 30 s, 55°C for 30 s, 72°C for 90 s; and 72°C for 5 min) (Yang et al., 2016). The *atc*A fragments (∼1100 bp), amplified from the sediment samples, were cloned in *Escherichia coli* (One Shot TOP10; Invitrogen) using the pGEM-T Easy Vector Systems (Promega). The *atcA* fragments were sequenced on an ABI 3730xI DNA Analyzer (Applied Biosystems) with BigDye terminator chemistry, according to the manufacturer’s instructions.

### Isolation of *Thauera* sp. Strain GDN1 from Estuarine Sediment Incubated with Testosterone and Nitrate

The denitrifying basic medium used for the isolation and routine cultivation of *Thauera* sp. strain GDN1 (strain GDN1 hereafter) contained 10 g of NaCl, 0.5 g of NH_4_Cl, 0.1 g of CaCl_2_⋅2H_2_O, 0.5 g of MgSO_4_⋅7H_2_O, 0.85 g of NaNO_3_ (10 mM), and 1 mM testosterone per liter. After autoclaving, this basic medium was supplemented with the following sterile chemicals: 50 mL of 1 M NaHCO_3_, 12.5 mL of 1 M KH_2_PO_4_, 1 mL of EDTA-chelated mixture of trace elements (Rabus and Widdel, 1995), 1 mL of selenite and tungstate solution (Tschech and Pfennig, 1984), and 1 mL of vitamin solution VL-7 (Pfennig, 1978). The final pH was adjusted to 6.5 with HCl. The sediment (0–5 cm depth; 100 g)–river water (900 mL) mixture was anaerobically incubated with 1 mM testosterone and 10 mM nitrate for 8 days. This testosterone-spiked mixture (1 mL) was subcultured in the denitrifying medium for two times. After the testosterone had been exhausted, the second sub-culture was serially diluted (10^-1^–10^-7^) in the denitrifying medium to obtain highly enriched cultures. The resulting cultures were spread on R2A agar (BD Difco) containing 1 mM testosterone and aerobically incubated at 28°C for 1 week. Two colonies were picked up from the agar plates and transferred to a denitrifying medium containing 1 mM testosterone and 10 mM acetate. The anaerobic degradation of testosterone in the denitrifying bacterial cultures was confirmed through HPLC. Purity was checked microscopically and by growth tests in liquid R2A medium or R2A agar. The 16S rRNA genes of the two isolated colonies capable of anaerobic testosterone degradation in the denitrifying medium were PCR-amplified using the bacterial 16S rRNA universal primers 27F (5′-AGAGTTTGATCCTGGCTCAG-3′) and 1492R (5′-GGTTACCTTGTTACGACTT-3′) (Lane, 1991), and the resulting PCR products were sequenced to verify the identity of the isolates.

To test the salinity tolerance of strain GDN1, this bacterial strain was anaerobically grown in denitrifying media containing testosterone (1 mM), acetate (10 mM), and different concentrations of NaCl (0–50 ppt). The cultures were sampled daily, and culture samples (0.5 mL) were centrifuged at 10,000 × *g* for 10 min. After centrifugation, the bacterial pellets were stored at -20°C before use. Proteins were extracted from the frozen pellets using B-PER Bacterial Cell Lysis Reagents (Thermo Fisher Scientific). The protein content in the samples was determined using BCA protein assay (Pierce BCA protein assay kit; Thermo Fisher Scientific), according to manufacturer’s instructions, with bovine serum albumin as the standard.

### Statistical Analysis

General statistical analyses (means ± SD) were performed using SigmaPlot 12.3 software. Bray–Curtis similarities for temporal changes of bacterial community structure spiked with testosterone or/and electron acceptors were visualized in two-dimensional plots using non-metric multidimensional scaling (nMDS) analysis. Taxonomic abundance data (Class level) obtained from Miseq sequencing was square-root transformed. 2-Dimensional (2D) stress value was indicated as good or poor representation in reduced dimensions among treated samples (Legendre and Legendre, 1998; Ramette, 2007). Significance testing for community structure under different treatments within same sediment sample was achieved using ANOSIM. All analyses were performed on software PRIMER-E v.6 (Clarke, 1993). Results are presented in Supplementary Figure S7.

## Results

### Anaerobic Testosterone Biodegradation in Estuarine Sediment under Nitrate-Reducing Conditions

**FIGURE 2:**
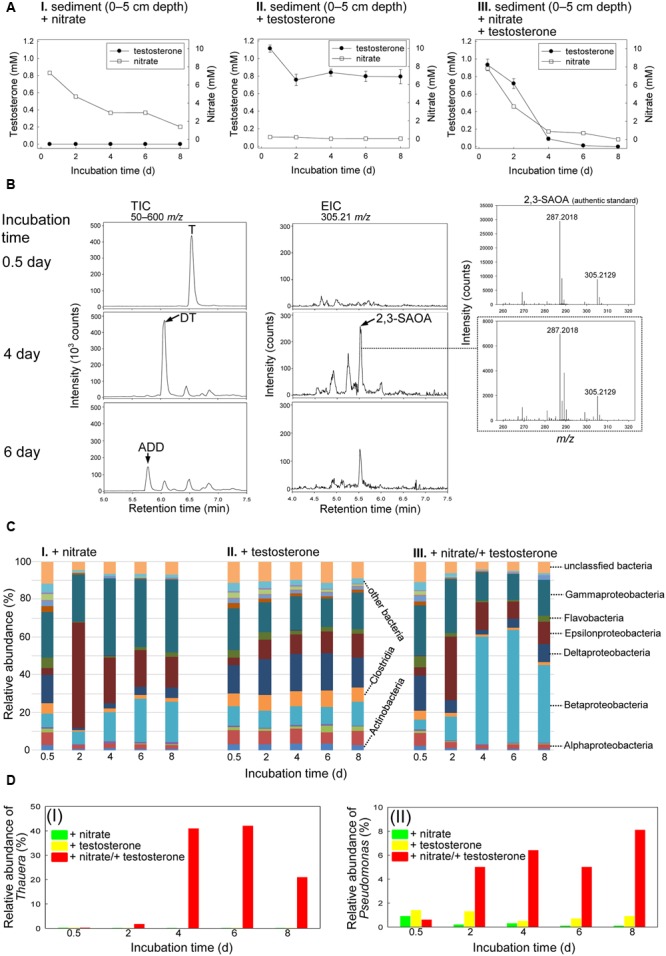
Anaerobic testosterone catabolism in subsurface layer sediment (0–5 cm depth)–river water mixture spiked with testosterone or nitrate or both. **(A)** The consumption of testosterone and nitrate in the sediment treatments. Data are shown as means ± SD of three experimental measurements. **(B)** UPLC–ESI–MS analysis of the ethyl acetate extracts of the sediment treatments. (Left panel) Total ion chromatograms of subsurface layer sediment–river water mixture spiked with testosterone and nitrate. (Middle panel) Extracted ion chromatograms for 2,3-SAOA (*m/z* = 305.21; expected retention time = 5.56 min) in subsurface layer sediment–river water mixture spiked with testosterone and nitrate. (Right panel) MS spectra of the authentic standard (top) and 2,3-SAOA extracted from the denitrifying sediment treatment. Abbreviations: ADD, androsta-1,4-diene-3,17-dione; DT, 1-dehydrotestosterone; T, testosterone. **(C)** Class-level phylogenetic analysis (Illumina MiSeq) revealed the temporal changes in the bacterial community structures in various sediment treatment samples. The non-metric multidimensional scaling (nMDS) analysis of the bacterial community structures in the treatments is shown in Supplementary Figure S7A. The further analyses of the community structures are shown in Supplementary Figure S8 and Tables S3–S5. **(D)** *Thauera* spp. (I) and *Pseudomonas* spp. (II) were enriched in subsurface layer sediment–river water mixture spiked with testosterone and nitrate.

The original Guandu subsurface layer sediment (0–5 cm depth)–river water mixture contained 1.3 nM androgens (mainly testosterone), 87 μM nitrate, 11 μM Fe^3+^, and 4.4 mM sulfate (Supplementary Table S1), with a salinity of 12 practical salinity units (PSU). The subsurface layer sediment–river water mixtures were spiked with testosterone (1 mM) or nitrate (10 mM) or both; then the spiked mixtures were incubated under anoxic conditions. Androgenic metabolites extracted from the sediment treatments were identified through UPLC–ESI–MS and HPLC–UV. Nitrate was slowly consumed in the sediment–river water mixture incubated without testosterone (**Figure 2AI**), which may have been due to the metabolism of unidentified organic compounds in the original sediment samples. Small quantities of testosterone were consumed in the absence of nitrate (**Figure 2AII**; see Supplementary Figure S1A for the HPLC analysis of the androgen metabolites). By contrast, in the presence of nitrate, testosterone was apparently consumed (**Figure 2AIII**). Testosterone was largely transformed to 1-dehydrotestosterone (DT) and androsta-1,4-diene-3,17-dione (ADD) under denitrifying conditions (**Figure 2B**), as confirmed through HPLC–UV (Supplementary Figure S1B). We employed extracted ion current (EIC) for *m/z* 305.21 (most dominant ion peak of 2,3-SAOA) to detect 2,3-SAOA in the denitrifying mixture. The UPLC retention time (5.56 min) and ESI–MS spectrum of the extracted ion were the same as those of the authentic standard (**Figure 2B**). This ring-cleaved metabolite was produced after a 4-day incubation, indicating that anaerobic testosterone degradation occurs under denitrifying conditions through the 2,3-*seco* pathway. Testosterone was exhausted and no ethyl acetate-extractable metabolites were apparently detected after the mixture was incubated with testosterone and nitrate for 8 days (**Figure 2AIII** and Supplementary Figure S1B).

Except for the unassigned and other (individual class with a relative percentage of <1%) bacteria, 16 classes were identified overall in the subsurface layer sediment–river water mixture. The initial (0.5-day incubation) bacterial community structures in the three treatments (spiked with nitrate alone, testosterone alone, and both nitrate and testosterone) were highly similar, and the abundant bacterial classes were Betaproteobacteria (5.3–10.5%), Gammaproteobacteria (23.2–26.9%), and Deltaproteobacteria (14.9–18.9%; **Figure 2C**). The members of Gammaproteobacteria were the most abundant bacteria in the initial sediment samples. In the anaerobic sediment incubated with testosterone alone (no exogenous nitrate), we did not observe remarkable temporal changes in the bacterial community structures (**Figure 2CII**). By contrast, in the presence of nitrate and testosterone, Betaproteobacteria were apparently enriched (57.3% abundance after 4 days; **Figure 2CIII**). Furthermore, we observed a considerable increase in the relative abundance of *Thauera* spp. (class Betaproteobacteria) in the denitrifying treatment incubated with testosterone (40.9% abundance after 4 days; **Figure 2DI**). The enrichment of *Thauera* spp. did not occur in the sediment–river water mixture incubated with nitrate or testosterone alone. Although the abundance of Gammaproteobacteria did not increase in the denitrifying sediment (**Figure 2CIII**), an apparent enrichment of *Pseudomonas* spp. (class Gammaproteobacteria) was observed (6.4% abundance after 4 days; **Figure 2DII**).

To gain rigorous evidence for the enrichment of the *Thauera* spp. population in the testosterone-treated sediment–river water mixtures, we performed a quantitative PCR study to examine the temporal changes in the 16S rRNA of *Thauera* spp. in different treatments. The abundance of the *Thauera* spp. genes in each sample was normalized by the total eubacterial 16S rRNA gene. The real-time quantitative PCR results were coherent with those of the metagenomic analysis. The relative abundance of the *Thauera* spp. 16S rRNA genes apparently increased after 4 days of incubation with testosterone and nitrate (Supplementary Figure S2).

**FIGURE 3:**
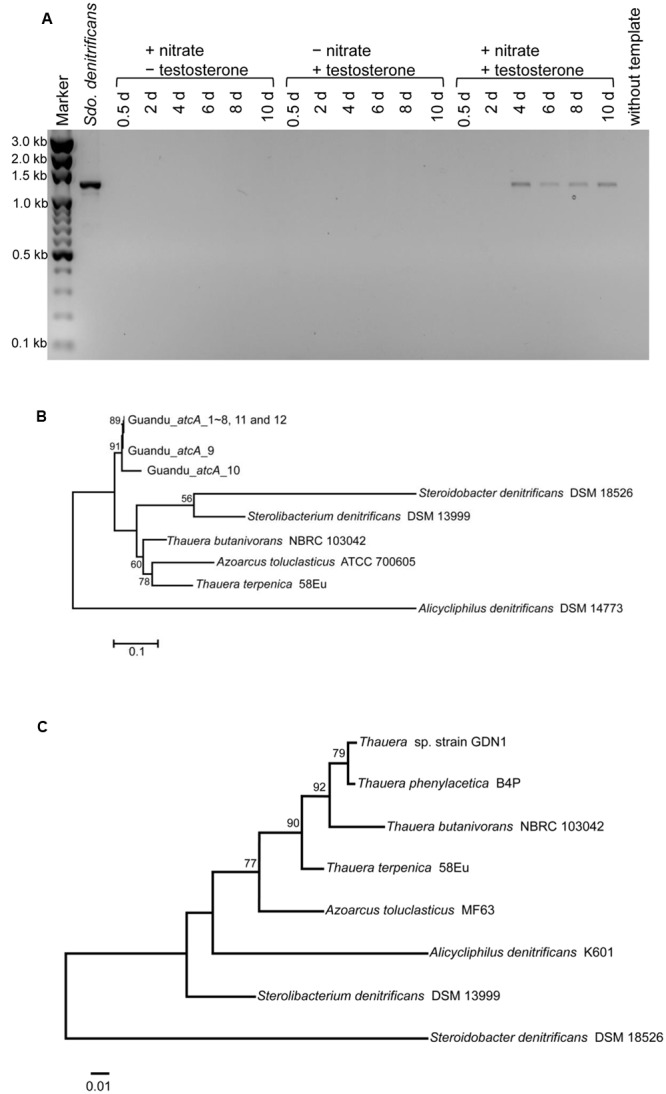
**(A)** Agarose gel electrophoresis revealed that *atcA*-like PCR products were detected only in subsurface layer sediment–river water mixture spiked with testosterone and nitrate. PCR products with the expected size of approximately 1200 bp were amplified from the androgen-degrading denitrifier, *Sdo. denitrificans*. The full-length gel is presented in Supplementary Figure S3. **(B)** Maximum Likelihood tree of *atcA* gene fragments obtained from the subsurface layer sediment–river water mixture incubated with testosterone and nitrate for 4 days. Refer to Supplementary Table S2 for individual *atcA* sequences. The gene encoding the large subunit (MhyADHL) of 3-hydroxycyclohexanone dehydrogenase from *Alicycliphilus denitrificans* served as an outgroup sequence. **(C)** The phylogenetic tree of 16S rRNA gene of strain GDN1. All sequences were aligned by MUSCLE. Both of the evolutionary histories were inferred by using the Maximum Likelihood method. Bootstrap values are based on 1000 replicates. Numbers shown around branches are bootstrap percentages for clades supported above the 50% level. Branch support was determined by bootstrapping 1000 times. The unit for each of the scale bar is nucleotide substitutions per site.

The *atcA*-specific degenerate primers were used for detecting this essential degradation gene in the sediment-river water mixtures. During denitrifying incubation with testosterone, PCR products of the expected size (∼1200 bp) were detected in the subsurface layer sediment–river water samples (**Figure 3A**). By contrast, the expected PCR products were not detected in the sediment-river water mixtures incubated with testosterone or nitrate alone. PCR products amplified from denitrifying sediment–river water mixture (4-day incubation with testosterone) were cloned in *E*. *coli*, and 12 clones were randomly selected for sequencing. The nucleotide sequences of Guandu_*atcA*1∼12 were highly similar (>95% sequence identity; see Supplementary Table S2 for individual sequences). The deduced amino acid sequences of all Guandu *atcA* fragments (Guandu_*atcA*1∼12) formed a distinct clade and were separated from those of the sludge isolates [*Sdo. denitrificans* (Fahrbach et al., 2008), *Stl. denitrificans* (Tarlera and Denner, 2003), *T. butanivorans* (Dubbels et al., 2009)] or bacterium isolated from aquifer sediment [*Azoarcus toluclasticus* (Song et al., 1999)] (**Figure 3B**).

### Isolation of Testosterone-Degrading Strain GDN1

**FIGURE 4:**
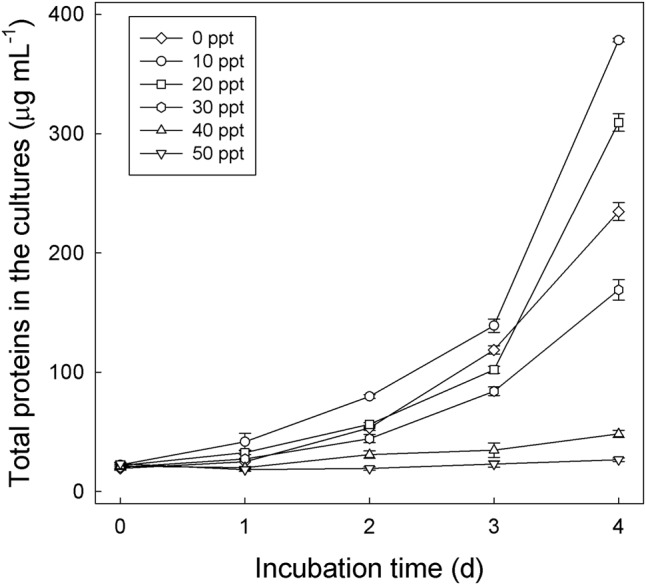
Salinity tolerance of *Thauera* sp. strain GDN1. Strain GDN1 was anaerobically grown in the denitrifying medium containing 1 mM testosterone, 10 mM acetate, and 0–50 ppt NaCl. Bacterial growth was measured as the total protein concentration in the cultures. Data are shown as means ± SD of three experimental measurements.

The sediment–river water mixture incubated with testosterone and nitrate for 8 days was serially transferred into a chemically defined medium containing 10 mM nitrate, 1 mM testosterone, and 1% (w/v) NaCl. The highly enriched cultures were spread on R2A agar containing 1 mM testosterone (aerobic incubation), and single colonies were selected for incubation with testosterone under denitrifying conditions. Subsequently, we isolated the testosterone-degrading denitrifier strain GDN1 (Guandu sediment incubated under nitrate-reducing conditions). Strain GDN1 can anaerobically degrade testosterone (1 mM) in the presence of acetate (10 mM) and nitrate (10 mM); however, it cannot use testosterone as sole carbon and energy source under denitrifying conditions. In addition, it cannot use Fe^3+^ or sulfate as alternative electron acceptor to degrade testosterone, even in the presence of 10 mM acetate. Strain GDN1 can anaerobically grow with testosterone and acetate under 0–30-ppt salinity, with an optimal growth under 10-ppt salinity (**Figure 4**). However, no apparent growth was observed under NaCl concentrations of >40 ppt. The *atcA* (Guandu_*atcA*1) and 16S rRNA (GDN1_16S rRNA) sequences of strain GDN1 are shown in Supplementary Table S2. Phylogenetic analysis of the 16S rRNA gene sequence showed that strain GDN1 is closely related to the testosterone-degrading denitrifier *T. terpenica* 58Eu (**Figure 3C**).

### Anaerobic Testosterone Metabolism in Estuarine Sediment under Iron-Reducing Conditions

**FIGURE 5:**
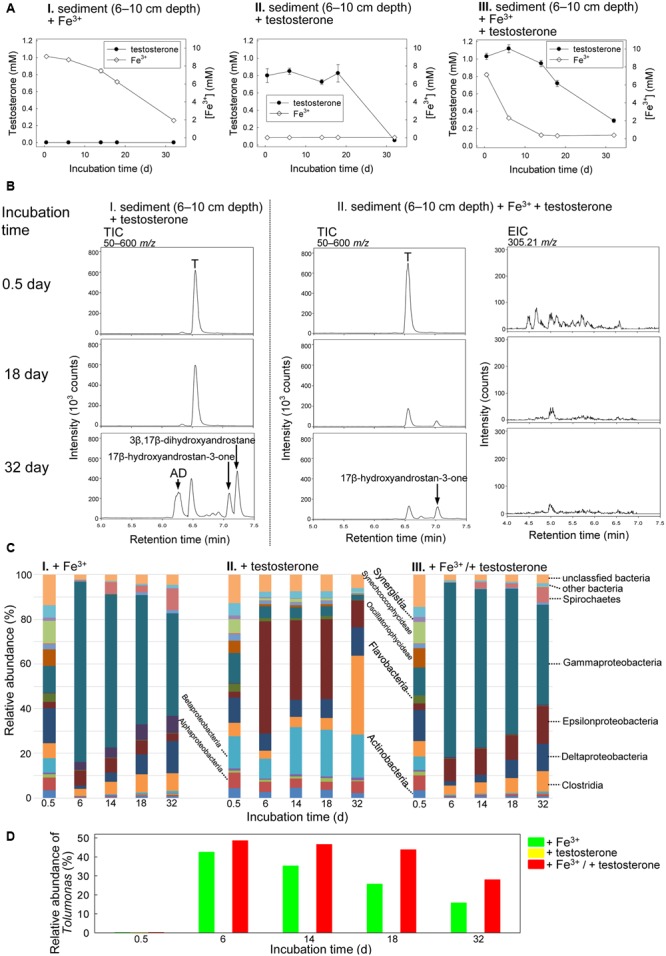
Anaerobic testosterone metabolism in middle layer sediment (6–10 cm depth)–river water mixture spiked with testosterone or Fe^3+^ or both. **(A)** Consumption of testosterone and Fe^3+^ in the sediment treatments. Data are shown as means ± SD of three experimental measurements. **(B)** UPLC–ESI–MS analysis of the ethyl acetate extracts of the sediment treatments. (Left panel) Total ion chromatograms of middle layer sediment–river water mixture spiked with testosterone alone. (Middle panel) Total ion chromatograms of middle layer sediment–river water mixture spiked with testosterone and Fe^3+^. (Right panel) Extracted ion chromatograms for 2,3-SAOA (m/z = 305.21; expected retention time = 5.56 min) in middle layer sediment–river water mixture spiked with testosterone and Fe^3+^. AD, androst-4-en-3,17-dione; T, testosterone. **(C)** Class-level phylogenetic analysis (Illumina MiSeq) revealed the temporal changes in the bacterial community structures in various sediment treatments. The nMDS analysis of the bacterial community structures in the treatments is shown in Supplementary Figure S7B. **(D)** Genus-level phylogenetic analysis revealed the temporal changes in the relative abundance of *Tolumonas* spp. in the sediment treatments.

The Guandu middle layer sediment (6–10 cm depth)–river water mixtures were incubated with testosterone (1 mM) or Fe^3+^ (10 mM) or both. The chemical properties of the original mixture (salinity = 15 PSU) are shown in Table S1. The ethyl acetate extracts were analyzed through UPLC–ESI–MS and HPLC–UV. Fe^3+^ was slowly consumed in the sediment–river water mixture incubated without exogenous testosterone (**Figure 5AI**), which may have been due to the metabolism of unidentified organic compounds. In the absence of additional Fe^3+^ (10 mM), testosterone was not apparently consumed within 18 days. However, after 32 days of anaerobic incubation, we observed anaerobic transformation of testosterone to various androgenic metabolites, including AD, 17β-hydroxyandrostan-3-one [retention time = 7.03; dominant ion peak ([M-H_2_O+H]^+^) = *m/z* 273.2329; predicted elemental composition, C_19_H_30_O_2_], and 3β,17β-dihydroxyandrostane [retention time = 7.22; dominant ion peak ([M–H_2_O+H]^+^) = *m/z* 275.2386; predicted elemental composition, C_19_H_32_O_2_] (**Figures 5AII,BI**). We used HPLC–UV as an alternative tool to detect the testosterone-derived metabolites, which were not ionized in the UPLC–ESI–MS conditions. We observed the accumulation of 50 ± 7 (*n* = 3) μM E1 and 112 ± 9 (= 3) μM E2, along with the residue of 54 ± 5 (*n* = 3) μM testosterone, after the anaerobic incubation of the middle layer sediment–river water mixture with testosterone for 32 days (Supplementary Figure S4A). These results show that testosterone was transformed even in the absence of exogenous electron acceptors. Estrogens were not produced from testosterone during the anaerobic incubation of the sediment–river water mixture in the presence of exogenous Fe^3+^ (Supplementary Figure S4B).

The presence of testosterone apparently accelerated Fe^3+^ consumption (**Figure 5AIII**). We noted testosterone consumption under iron-reducing conditions (**Figures 5AIII,BII**). After 32 days of incubation under iron-reducing conditions, the peaks corresponding to testosterone and its derivatives were apparently reduced (**Figure 5BII** and Supplementary Figure S4B). However, the ring-cleaved product, 2,3-SAOA (expected UPLC retention time = 5.56 min), was not detected in the sediment sample incubated with testosterone and Fe^3+^ (**Figure 5BII**). Furthermore, *atcA*-like genes were not detected in the sediment sample incubated with testosterone and Fe^3+^ (Supplementary Figure S5A). The temporal changes in the bacterial community structures in the middle layer sediment–river water samples were also analyzed. The initial bacterial community structures in the three treatments (incubated with Fe^3+^ alone, testosterone alone, and both Fe^3+^ and testosterone) were highly similar: Actinobacteria (3.4–4.3%), Alphaproteobacteria (5.7–7.1%), Betaproteobacteria (6.1–14.6%), Gammaproteobacteria (12.0–13.6%), Deltaproteobacteria (11.3–15.8%), Clostridia (6.0–7.1%), Flavobacteria (3.9–6.3%), and two cyanobacterial classes, Oscillatoriophycideae (5.5–8.5%), and Synechcoccophycideae (6.3–10.1%; **Figure 5C**). We observed similar patterns of temporal changes in the bacterial community structures in the middle layer sediment–river water mixture incubated with Fe^3+^ alone (**Figure 5CI**) and that incubated with both Fe^3+^ and testosterone (**Figure 5CIII**), with a significant increase in the relative abundance of Gammaproteobacteria in both treatments. At the genus level (**Figure 5D**), we observed an apparent increase of the relative abundance of *Tolumonas* spp. (class Gammaproteobacteria) in the middle layer sediment–river water sample incubated with both Fe^3+^ and testosterone (from 0.2% at day 0.5 to 43.6% at day 18). Although an increase in the abundance of *Tolumonas* spp. also occurred in the sediment–river water mixture incubated with Fe^3+^ alone for 6 days, that abundance apparently decreased with time later (**Figure 5D**).

### Anaerobic Testosterone Metabolism in Estuarine Sediment Spiked with Testosterone and Sulfate

**FIGURE 6:**
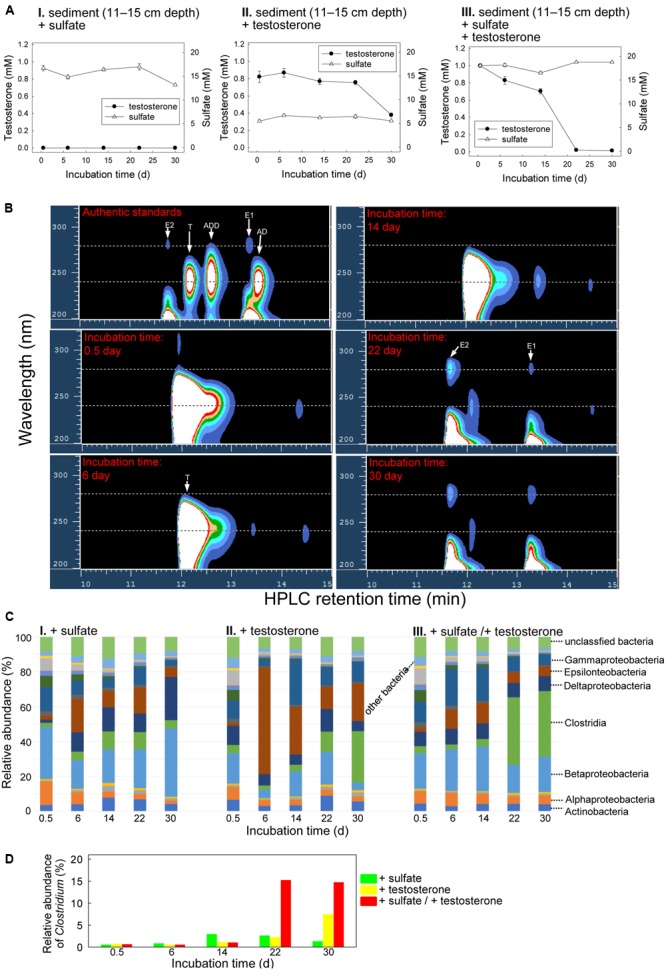
Anaerobic testosterone metabolism in bottom layer sediment (11–15 cm depth)–river water mixture spiked with testosterone or sulfate or both. **(A)** Consumption of testosterone and sulfate in the sediment treatments. Data are shown as means ± SD of three experimental measurements. **(B)** HPLC–UV analysis of the ethyl acetate extracts of bottom layer sediment–river water mixture spiked with testosterone and sulfate. During anaerobic incubation, estrogens accumulated in the treatment samples. AD, androst-4-en-3,17-dione; ADD, androsta-1,4-diene-3,17-dione; E1, estrone; E2, 17β-estradiol; T, testosterone. **(C)** Class-level phylogenetic analysis (Illumina MiSeq) revealed the temporal changes in the bacterial community structures in various sediment treatment samples. The nMDS analysis of the bacterial community structures in the treatments is shown in Supplementary Figure S7C. **(D)** Genus-level phylogenetic analysis revealed the temporal changes in the relative abundance of *Clostridium* spp. in the testosterone-spiked sediment treatments.

The Guandu bottom layer sediment (11–15 cm depth)–river water mixtures were incubated with testosterone or sulfate or both. The chemical properties of the original mixture (salinity = 16 PSU) are shown in Supplementary Table S1. Sulfate (∼5 mM; **Figure 6AII**) was detected in the original Guandu sediment–river water mixture. Regardless of the presence or absence of additional sulfate (10 mM), we observed the transformation of testosterone to E1 and E2 during anaerobic incubation (**Figure 6B** and Supplementary Figure S6). After the anaerobic incubation of the bottom layer sediment–river water mixture with exogenous testosterone and sulfate for 30 days, 980 ± 17 (*n* = 3) μM testosterone was consumed, along with the production of 79 ± 5 (*n* = 3) μM E1 and 94 ± 8 μM (*n* = 3) E2 (**Figure 6B**). Sulfate was not consumed in these treatments (**Figure 6A**). Furthermore, *atcA*-like genes were not detected in bottom layer sediment samples spiked with testosterone or sulfate or both (Supplementary Figure S5B).

The temporal changes in the bacterial community structures from the bottom layer sediment–river water samples were also analyzed. The bacterial community structures in the initial stages of the three treatments were highly similar, mainly composed of Betaproteobacteria (17.4–29.9%; **Figure 6C**). However, the abundance of Betaproteobacteria did not apparently increase during anaerobic incubation in the three treatments. By contrast, an apparent increase of Clostridia members was observed in the sediment samples incubated with testosterone (**Figure 6CII,III**), regardless of the addition of sulfate (10 mM). This is consistent with the enrichment of *Clostridium* spp. in the testosterone-spiked treatments (**Figure 6D**).

## Discussion

Knowledge regarding the microbial androgen metabolism in anaerobic sediments is extremely limited. Thus far, the 2,3-*seco* pathway is the only known anaerobic androgen biodegradation pathway. This pathway includes some unique intermediates, such as 2,3-SAOA, which are not observed in other catabolic pathways. In addition, the genes corresponding to this pathway (e.g., *atcA*) have been found in the genomes of a few denitrifying bacteria from the Betaproteobacteria and Gammaproteobacteria classes (Yang et al., 2016). Based on sediment chemical profiles and the vertical distribution of anaerobic bacterial taxa (Fan et al., 2006, 2012), we separated the sediment cores into three sections: subsurface layer (0–5 cm depth), middle layer (6–10 cm depth), and bottom layer (11–15 cm depth). Chemical analyses of the original sediment–river water mixtures confirmed that nitrate, Fe^3+^, and sulfate are most abundant in the subsurface layer, middle layer, and bottom layer, respectively. The androgen content in the original Guandu sediment–river water mixtures was 1.2–1.7 nM, with testosterone as the major constituent (Supplementary Table S1). The androgens detected in the Guandu sediments may have been discharged from the Dihua Sewage Treatment Plant [effluent androgen content = approximately 0.9 nM androgens (Yang et al., 2016)] or were produced from sterols *in situ*. Sterols in the river water and sediments can be transformed into androgens by microorganisms (Jenkins et al., 2001, 2003).

The characteristic metabolite 2,3-SAOA and the essential catabolic gene *atcA* of the 2,3-*seco* pathway were detected in the subsurface layer sediment spiked with testosterone and nitrate, thus strongly demonstrating that testosterone can be efficiently removed from anaerobic sediment by denitrifying bacteria through the 2,3-*seco* pathway. The microbial community analysis suggested that denitrifying proteobacteria, including *Thauera* spp. and *Pseudomonas* spp., are major players for this anaerobic bioprocess.

By using similar approaches, Yang et al. (2016) identified *Thauera* spp. as potential testosterone degraders in the denitrifying sewage collected from a wastewater treatment plant, but could not isolate the testosterone degraders. In the present study, we isolated strain GDN1 from the denitrifying sediment; this strain can tolerate a broad range of salinity (0–30 ppt), reflecting the fluctuating salinity (5–22 ppt) in Guandu (Fan et al., 2006; Hsieh et al., 2010). The 16S rRNA gene of strain GDN1 showed high sequence similarity (99%) to several *Thauera* spp, including *T. aminoaromatica, T. chlorobenzoica, T. phenylacetica*, and *T. selenatis*. The *atcA* sequence of strain GDN1 was highly similar (>95% nucleotide sequence identity) to other *atcA* genes (Guandu_*atcA*2∼12) amplified from the subsurface layer sediment–river water mixture incubated with both testosterone and nitrate for 4 days, suggesting that we isolated a major testosterone degrader in this sediment treatment. *Thauera* spp. are common in anaerobic sediments. For instance, *T. chlorobenzoica* strains 4FB1 and 4FB2 as well as *T. aromatica* strain 3CB2 were isolated from estuarine sediments (Song et al., 2001). In addition, the benzoyl-CoA reductase gene, the essential catabolic gene for anaerobic degradation of aromatic compounds, associated with *Thauera* spp. was detected in freshwater and estuarine sediments (Song and Ward, 2005). Strain GDN1 cannot use testosterone as a sole source of carbon and energy. In addition, androgens are typically present at low concentrations (<1 μg/L) in the environment. Therefore, in natural habitats (e.g., estuarine sediments), strain GDN1 may not depend on androgens as its major carbon source. Strain GDN1 (this study) and *Thauera terpenica* 58Eu (Yang et al., 2016) depend on acetate for anaerobic testosterone degradation. In anaerobic sediments, volatile fatty acids (e.g., acetate) serve as both the major end-products of fermentation and as substrates for microbial degradation. For instance, acetate is the principal substrate for sulfate-reducing bacteria in marine sediments (Sørensen et al., 1981). Chemical analyses in the estuarine sediments collected from the Tamar Estuary, United Kingdom indicated that acetate concentrations were high (∼25 μM) near the surface (0–3 cm depth) and then decreased with sediment depth (Wellsbury and Parkes, 1995). In the subsurface layer of estuarine sediments, acetate and other volatile fatty acids may enhance the anaerobic testosterone degradation by the denitrifying *Thauera* spp.

Although an increase in the relative abundance of *Pseudomonas* spp. was observed in the sediment–river water mixture incubated with both testosterone and nitrate, we were not able to amplify any *atcA*-like genes from *Pseudomonas* spp. Furthermore, we were not able to isolate any *Pseudomonas* spp. capable of anaerobic testosterone degradation in any of our cultivation conditions and growth media. Thus far, *Pseudomonas* spp. have not been reported as androgen-degrading denitrifiers. Therefore, based only on the relative increase in abundance, we cannot confirm the testosterone degradation capability of *Pseudomonas* spp. in the denitrifying sediments. These bacteria might be involved indirectly in the degradation process by feeding on testosterone degradation intermediates released by the actual testosterone degraders. Alternatively, *Pseudomonas* spp. may be flourishing through co-metabolism of other carbon substrates in the sediment–river water mixture.

Bacteria may adopt different biochemical pathways and enzymes to import and transform testosterone, depending on the available substrate concentration. To enable enrichment of the potential testosterone degraders, the estuarine sediments were incubated with a high concentration of testosterone (1 mM), which is approximately six orders of magnitude higher than that detected in the Guandu sediments. Accordingly, we cannot exclude that ecologically relevant pathways other than the 2,3-*seco* pathway might be involved in anaerobic testosterone degradation. Moreover, it remains unclear whether *Thauera* spp. and *Pseudomonas* spp. are responsible for *in situ* androgen degradation in the estuarine sediments.

The results of these experiments show, for the first time, that testosterone can be degraded, at least partially, under iron-reducing conditions. In the ethyl acetate extracts of the middle layer sediment–river water mixture incubated with testosterone and Fe^3+^, we detected apparent substrate consumption; no testosterone-derived metabolites were accumulated. However, our current data cannot fully support the complete degradation of testosterone to CO_2_ under iron-reducing conditions. We cannot exclude that testosterone was partially degraded into hydrophilic compounds, which remained in the aqueous phase after liquid–liquid partition. A recent study (Landry et al., 2017) suggested that recalcitrant steroids in deep-ocean may be partially degraded into more refractory products. Stoichiometric analyses have been widely applied to the biodegradation of bacterial cultures (Wang et al., 2014). However, this analysis is not feasible for environmental samples containing complex microbial communities and unidentified organic compounds. Here, testosterone degradation occurred in the sediment incubated under iron-reducing conditions; however, we did not observe any bacteria specifically enriched in the iron-reducing treatment, possibly because (i) various bacteria were capable of degrading testosterone under iron-reducing conditions, (ii) testosterone degradation is a cooperative bioprocess performed by various microorganisms, and (iii) partial testosterone degradation cannot support growth of the corresponding microorganism(s). We observed a relative increase in the number of *Tolumonas* spp. in the sediment incubated with testosterone and Fe^3+^, suggesting that *Tolumonas* spp. might play a role in anaerobic testosterone metabolism under iron-reducing conditions. The physiology and metabolism of *Tolumonas* spp. is largely unknown. The most studied species, *T. aurensis*, was isolated from the anaerobic sediments of a freshwater lake (Fischer-Romero et al., 1996). This bacterium can use various sugars as its carbon source under anoxic conditions. Under iron-reducing conditions, *Tolumonas* spp. may have used various organic compounds, including testosterone and those in the original sediment sample, as its carbon and energy sources. This postulation is consistent with the observed temporal increase in the abundance of *Tolumonas* spp. in the sediment incubated with Fe^3+^ but without testosterone. The role of *Tolumonas* spp. in testosterone metabolism under iron-reducing conditions warrants further investigation. Nevertheless, our chemical data suggest that anaerobic testosterone degradation occurs under iron-reducing conditions. In the iron-reducing sediments, we could not detect the signature metabolites and characteristic catabolic gene (*atcA*) of the 2,3-*seco* pathway, suggesting that testosterone biodegradation under iron-reducing conditions might be mediated by yet unknown catabolic pathways. In future physiological and metabolic studies, the isolation of the testosterone degraders under iron-reducing conditions is warranted.

Although abundant in the original sediment–river water samples, sulfate (∼5 mM) was not apparently consumed in the testosterone-spiked sediment treatments. Our data thus suggest that testosterone degradation does not occur under sulfate-reducing conditions. A highlight of this study is the anaerobic transformations of testosterone into estrogens. The accumulation of estrogens (up to 173 μM) only occurred in the testosterone-spiked sediments. In addition, testosterone transformation was not detected in the autoclaved sediments. These results strongly suggest microbial transformation of testosterone to estrogens in the strictly anaerobic sediments. It is known that animals produce estrogens from androgens and this aerobic bioprocess is catalyzed by aromatase, a monooxygenase-containing protein complex (Czajka-Oraniec and Simpson, 2010). The aerobic transformation of androgens to estrogens involves three successive hydroxylations of the 19-methyl group of androgens (Cole et al., 1990). In contrast to the aerobic transformations of androgens to estrogens in animals, the transformation of testosterone to estrogens by microorganisms in the estuarine sediment–river water mixtures is strictly anaerobic. This anaerobic transformation cannot be mediated by aromatase-like enzymes, which require molecular oxygen as a co-substrate. We noted that the anaerobic transformation of testosterone to estrogens was accompanied by a significant enrichment of Clostridia (**Figures 5CII, 6CII,III**). So far, it remains unclear whether *Clostridium* spp. play a role in this anaerobic biotransformation. Moreover, considering the disappearance of testosterone (∼800 μM) from the sediment treatments, we cannot exclude the possibility that the enrichment of Clostridia members might be due to fermentative degradation of testosterone. To understand the anaerobic transformation of testosterone to estrogens at the molecular level, the involved microorganisms should be isolated from the anaerobic sediment and further characterized.

## Conclusion

Thus far, little is known about the biochemical mechanisms of steroid biodegradation operating in the oxygen-limited environments. Using the LC-MS based metabolite profile analysis, we identified the testosterone catabolic pathways functioning in the testosterone (1 mM)-spiked estuarine sediments. We also demonstrated that under laboratory conditions, bacteria could use nitrate or Fe^3+^ as alternative electron acceptors to oxidize testosterone in estuarine sediment. In the Guandu sediment, nitrate and denitrifying bacteria are largely distributed in the subsurface layer (0–5 cm depth). In this sediment layer, androgens produced and released from the aerobic regions bind to sediment particles through physical sorption (Kim et al., 2007) and then are efficiently degraded by denitrifying bacteria such as *Thauera* spp. through the 2,3-*seco* pathway. Although testosterone degradation also occurs under iron-reducing conditions, it proceeds more gradually than that under denitrifying conditions. In strictly anaerobic sediment, testosterone biodegradation is not associated with sulfate reduction. Our data thus suggest that in the estuarine sediment, androgens are mainly degraded by denitrifying bacteria in the subsurface layer.

## Author Contributions

Y-RC conceived of the study. C-JS, C-HW, and I-TL performed the androgen metabolites analysis. Y-LC and WI performed the metagenomic analysis and PCR-based functional assays. SW performed the statistical analyses. C-JS and I-TL isolated and characterized the testosterone-degrading bacterium. WI and Y-RC wrote the manuscript. All authors have read and approved this manuscript.

## Conflict of Interest Statement

The authors declare that the research was conducted in the absence of any commercial or financial relationships that could be construed as a potential conflict of interest.

## Footnotes

1 http://support.illumina.com/content/dam/illumina-support/documents/documentation/chemistry_documentation/16s/16s-metagenomic-library-prep-guide-15044223-b.pdf

2 http://rdp.cme.msu.edu/classifier/
