# Supplementary material for: Biochemical Mechanisms and Microorganisms Involved in Anaerobic Testosterone Metabolism in Estuarine Sediments
Source: Front Microbiol. 2017 Aug 11;8:1520. doi: 10.3389/fmicb.2017.01520 (PMC5554518; doi:10.3389/fmicb.2017.01520)
Supplement: Supplementary file 4 [file Table_5.pdf]

Table S5. The sequences of OTUs affiliated to the genus *Thauera*. Relative abundance of the following OTUs is more than 1% in at least one sediment sample.

>OTU7
TGGGGAATTTTGGACAATGGGCGCAAGCCTGATCCAGCCATGCCGCGTGAGT
GAAGAAGGCCTTCGGGTTGTAAAGCTCTTTCGGCCGGGAAGAAATCGTGTTC
TCTAACATAGGACATGGATGACGGTACCGGACTAAGAAGCACCGGCTAACTA
CGTGCCAGCAGCCGCGGTAATACGTAGGGTGCGAGCGTTAATCGGAATTACT
GGGCGTAAAGCGTGCGCAGGCGGTTTTGTAAGACAGATGTGAAATCCCCGGG
CTTAACCTGGGAACTGCGTTTGTGACTGCAAGGCTAGAGTACGGCAGAGGGG
GGTGGAATTCCTGGTGTAGCAGTGAAATGCGTAGAGATCAGGAGGAACACCG
ATGGCGAAGGCAGCCCCCTGGGCCTGTACTGACGCTCATGCACGAAAGCGTG
GGGA GCAAACA

>OTU307
TGGGGAATTTTGGACAATGGGGGCAACCCTGATCCAGCCATGCCGCGTGAGT
GAAGAAGGCCTTCGGGTTGTAAAGCTCTTTCAGCCGGGAAGAAAACGCACTC
TCTAACATAGGGTGTGGATGACGGTACCGGAAGAAGAAGCACCGGCTAACTA
CGTGCCAGCAGCCGCGGTAATACGTAGGGTGCGAGCGTTAATCGGAATTACT
GGGCGTAAAGGGTGCGCAGGCGGTTTTGTAAGACAGATGTGAAATCCCCGGG
CTTAACCTGGGAACTGCGTTTGTGACTGCAAGGCTAGAGTACGGCAGAGGGG
GGTGGAATTCCGCGTGTAGCAGTGAAATGCGTAGATATGCGGAGGAACACCG
ATGGCGAAGGCAACCCCCTGGGCCTGTACTGACGCTCATGCACGAAAGCGTG
GG GAGCAAACA
