# Supplementary material for: Biochemical Mechanisms and Microorganisms Involved in Anaerobic Testosterone Metabolism in Estuarine Sediments
Source: Front Microbiol. 2017 Aug 11;8:1520. doi: 10.3389/fmicb.2017.01520 (PMC5554518; doi:10.3389/fmicb.2017.01520)
Supplement: Supplementary file 5 [file Presentation_1.pdf]

Table S1. Chemical characteristics of original sediment–river water mixtures (not spiked with testosterone or electron acceptors).

	Sediment-river water mixtures		
Chemical properties	Sediment (0–5 cm depth; 100 g) + river water (900 mL)	Sediment (6–10 cm depth; 100 g) + river water (900 mL)	Sediment (11–15 cm depth; 100 g) + river water (900 mL)
T (nM)*	1.2 ± 0.1	1.4 ± 0.2	1.7 ± 0.2
DT (nM)	ND	ND	ND
AD (nM)	0.1 ± 0.0	ND	ND
ADD (nM)	ND	ND	ND
Nitrate (μM)	87 ± 6	45 ± 2	37 ± 3
Fe^{3+} (μM)	11 ± 2	27 ± 3	24 ± 2
Sulfate (mM)	4.4 ± 0.3	4.8 ± 0.2	5.1 ± 0.4

*Abbreviations: T, testosterone; DT, 1-dehydrotestosterone; AD, androst-4-en-3,17-dione; ADD, androsta-1,4-diene-3,17-dione; ND, not detected.

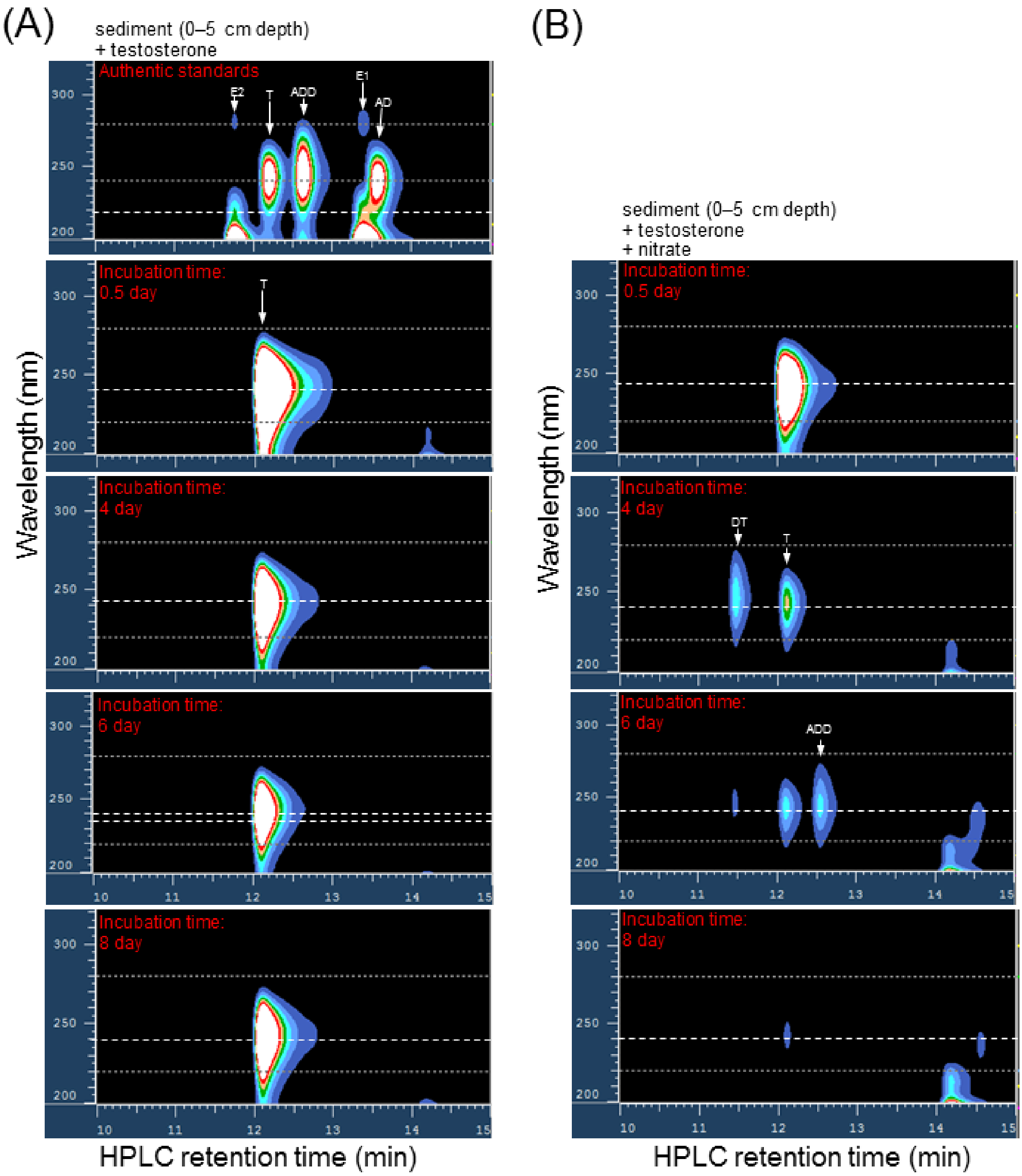

Figure S1. HPLC–UV analysis of the ethyl acetate extracts of subsurface layer sediment (0–5 cm)-river water mixture spiked with testosterone alone (A) and with both testosterone and nitrate (B). Abbreviations: AD, androst-4-en-3,17-dione; ADD, androsta-1,4-diene-3,17-dione; E1, estrone; E2, 17β-estradiol; T, testosterone.

(A)

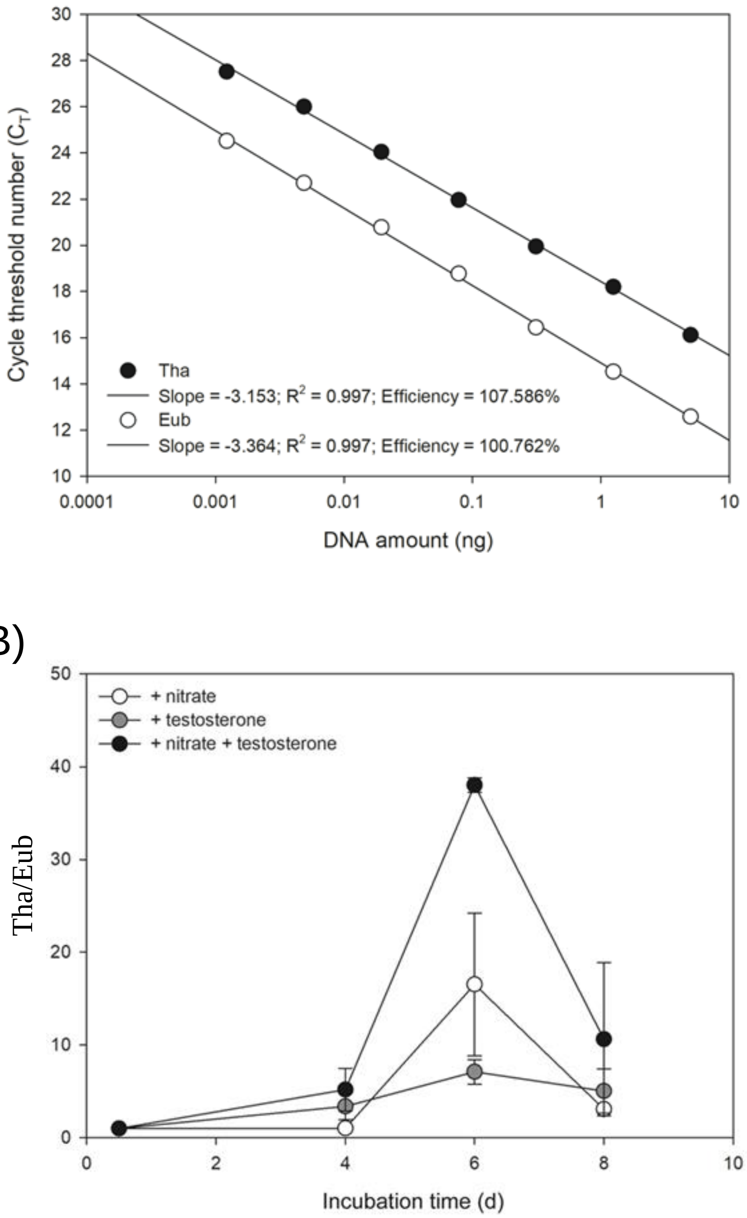

Figure S2. Real-time quantitative PCR. (A) Standard curves obtained using primer pairs Tha (solid circle) and Eub (open circle). Four fold serial dilutions of the genomic DNA of *Thauera* sp. strain GDN1 were used as the template DNA. Linear regressions were calculated using the dots of each series. (B) Temporal changes in the 16S RNA gene copies of *Thauera* spp. in subsurface layer sediment (0–5 cm)-river water mixture spiked with testosterone or nitrate or both. Specific primer pair Tha (this study) [forward primer (Tha-for): 5′–ACGTGAAGTCGGAATCGCTAGT–3′ and reverse primer (Tha-rev): 5′–AACCCACTCCCATGGTGTGA–3′] and general primer pair Eub [Muyzer *et al.*, (1993). *Appl. Environ. Microbiol.* 59, 695–700.] [forward primer (341F): 5′–CCTACGGGAGGCAGCAG–3′ and reverse primer (534R): 5′– ATTACCGCGGCTGCTGGC–3′] were used to amplify the 16S rRNA gene of *Thauera* spp. and total eubacterial population, respectively. Three replicates of real-time quantitative PCR experiments were performed using a QuantStudio 5 Real-Time PCR System (Applied Biosystems). The PCR mixture (20 µL) contained 10 µL of Power SYBR Green PCR master mix (Applied Biosystems), 0.1 µM for each primer, and 10 ng of environmental DNA. The thermal cycling conditions consisted of an initial denaturation step of 95 °C for 10 min, followed by 40 cycles of 95 °C for 15 s and 60 °C for 60 s. The primer pair Tha were derived from the sequence consensus of *Thauera* spp. in Fig. 3C. Data are shown as the mean ± SD of three experimental measurements.

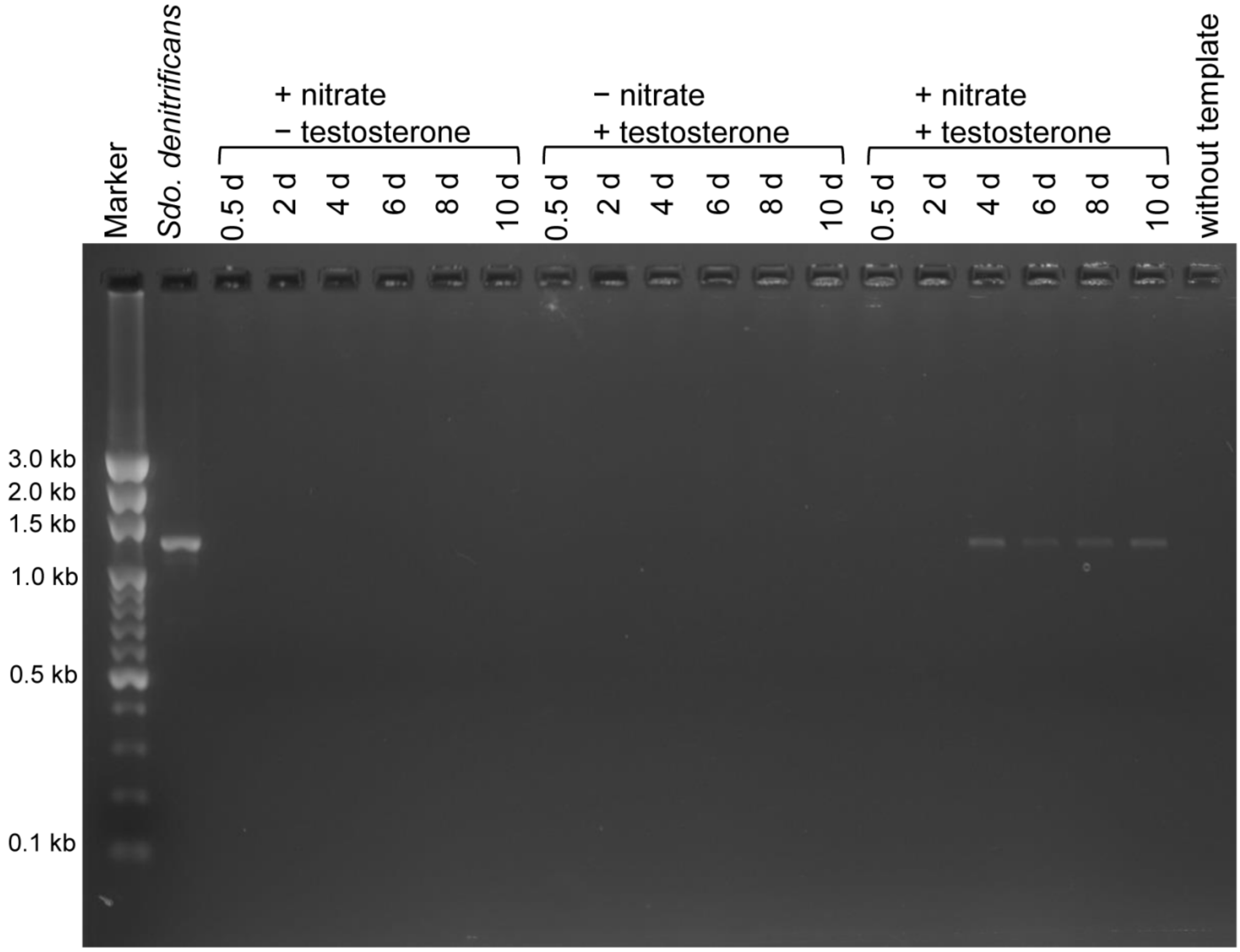

Figure S3. The original full-length agarose gel for the cropped gel image shown in Fig. 3A.

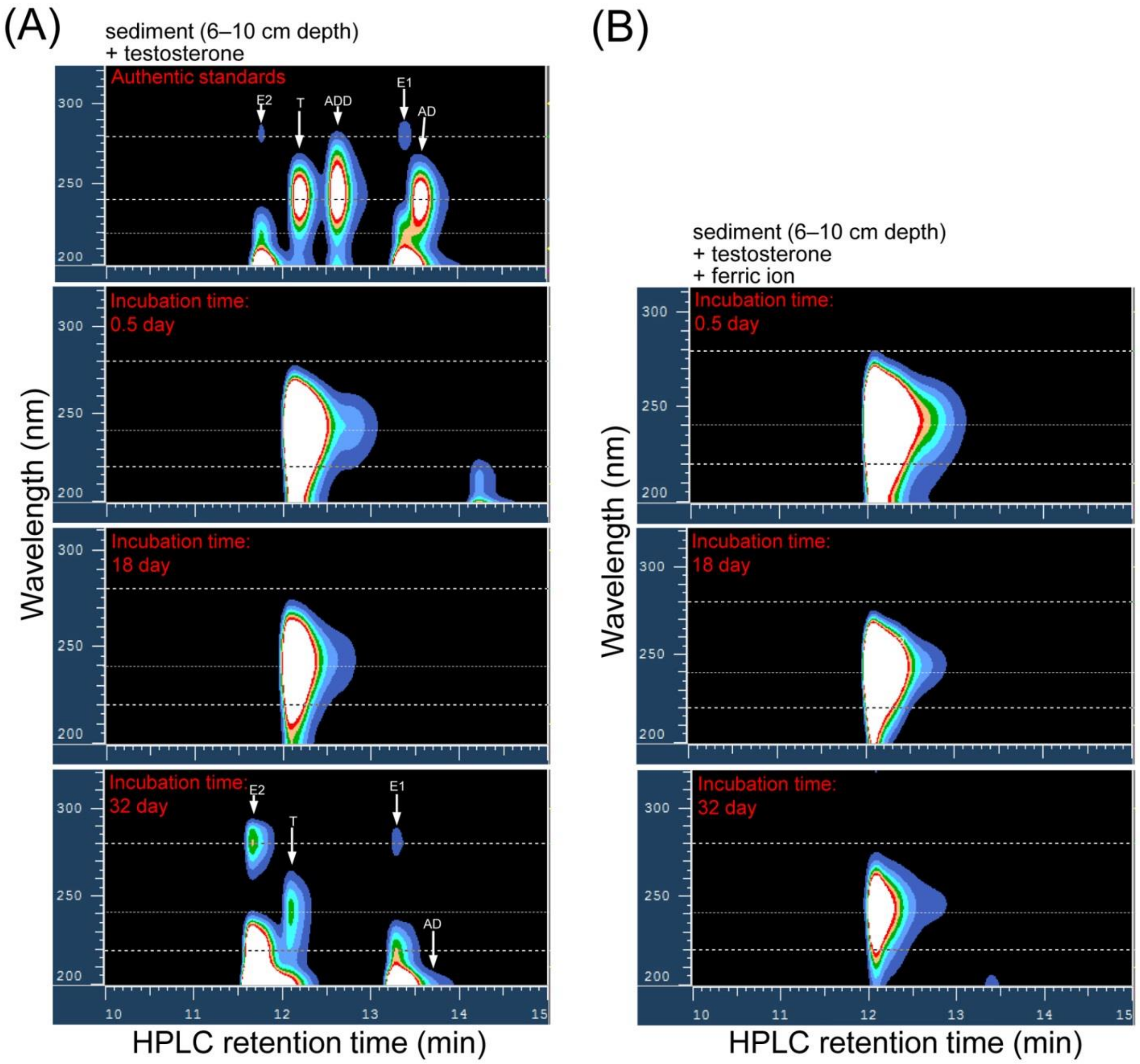

Figure S4. HPLC–UV analysis of the ethyl acetate extracts of middle layer sediment (6–10 cm)–river water mixture spiked with testosterone alone (A) and with both testosterone and Fe^{3+} (B). Abbreviations: AD, androst-4-en-3,17-dione; ADD, androsta-1,4-diene-3,17-dione; E1, estrone; E2, 17β-estradiol; T, testosterone.

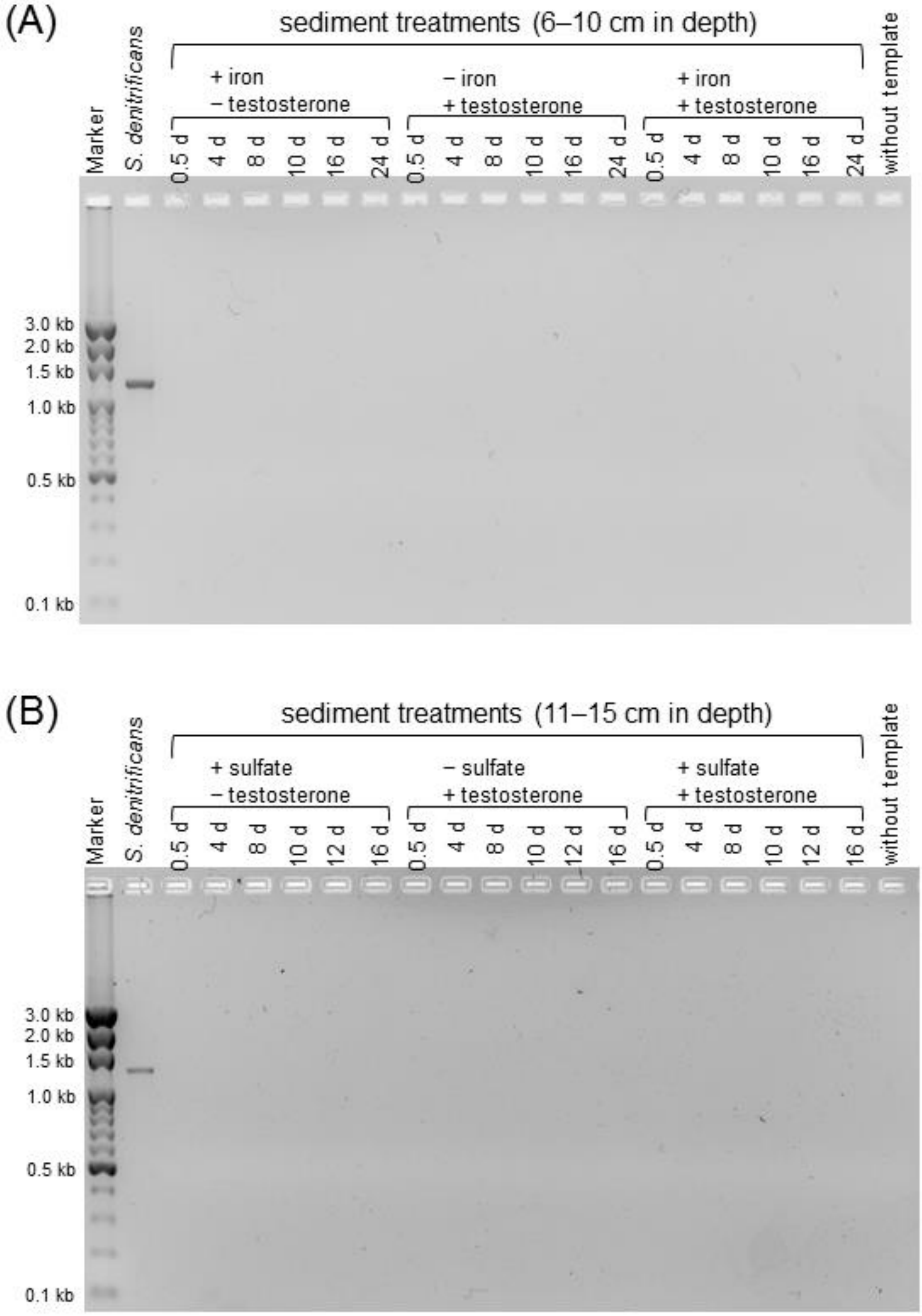

Figure S5. (A) Agarose gel electrophoresis revealed that *atcA*-like PCR products were not detected in middle layer sediment–river water mixture spiked with testosterone or Fe^{3+} or both. (B) Agarose gel electrophoresis showed that *atcA*-like PCR products were not detected in bottom layer sediment–river water mixture spiked with testosterone or sulfate or both. PCR products with the expected size of approximately 1200 bp were amplified from the androgen-degrading denitrifier, *Steroidobacter denitrificans* DSM 18526.

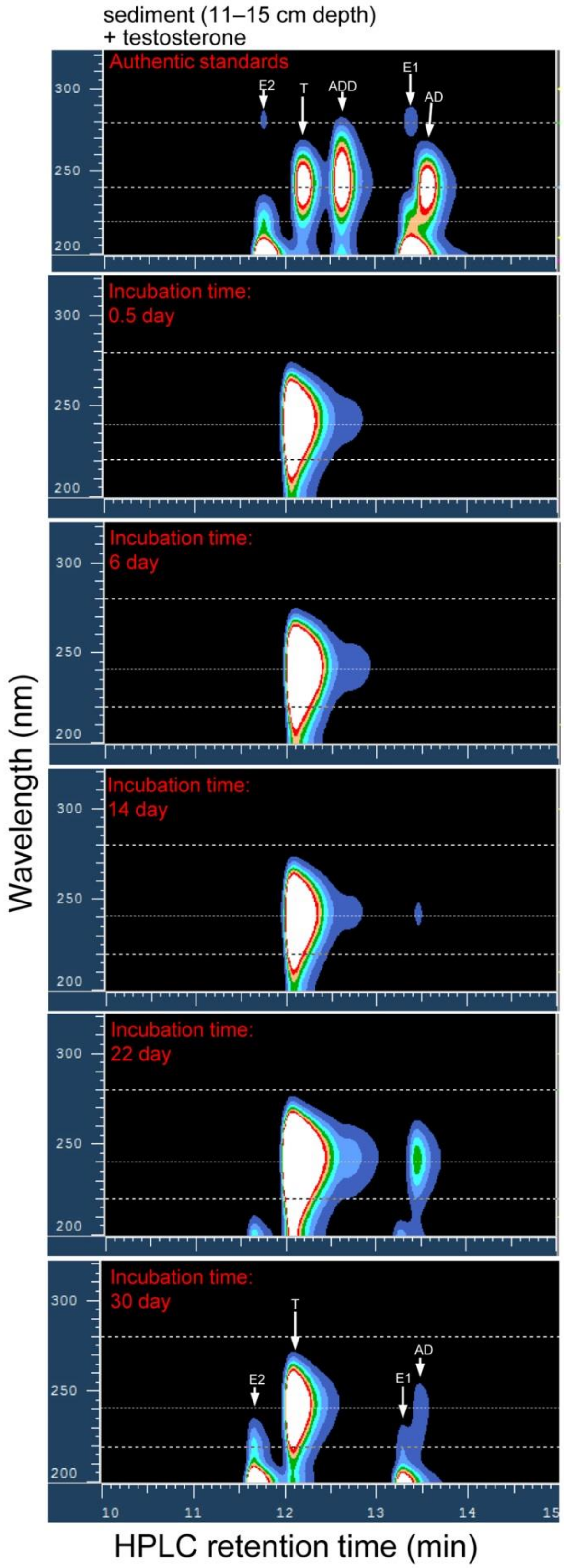

Figure S6. HPLC–UV analysis of the ethyl acetate extracts of bottom layer sediment (11–15 cm)–river water mixture spiked with testosterone alone. Abbreviations: AD, androst-4-en-3,17-dione; ADD, androsta-1,4-diene-3,17-dione; E1, estrone; E2, 17β-estradiol; T, testosterone.

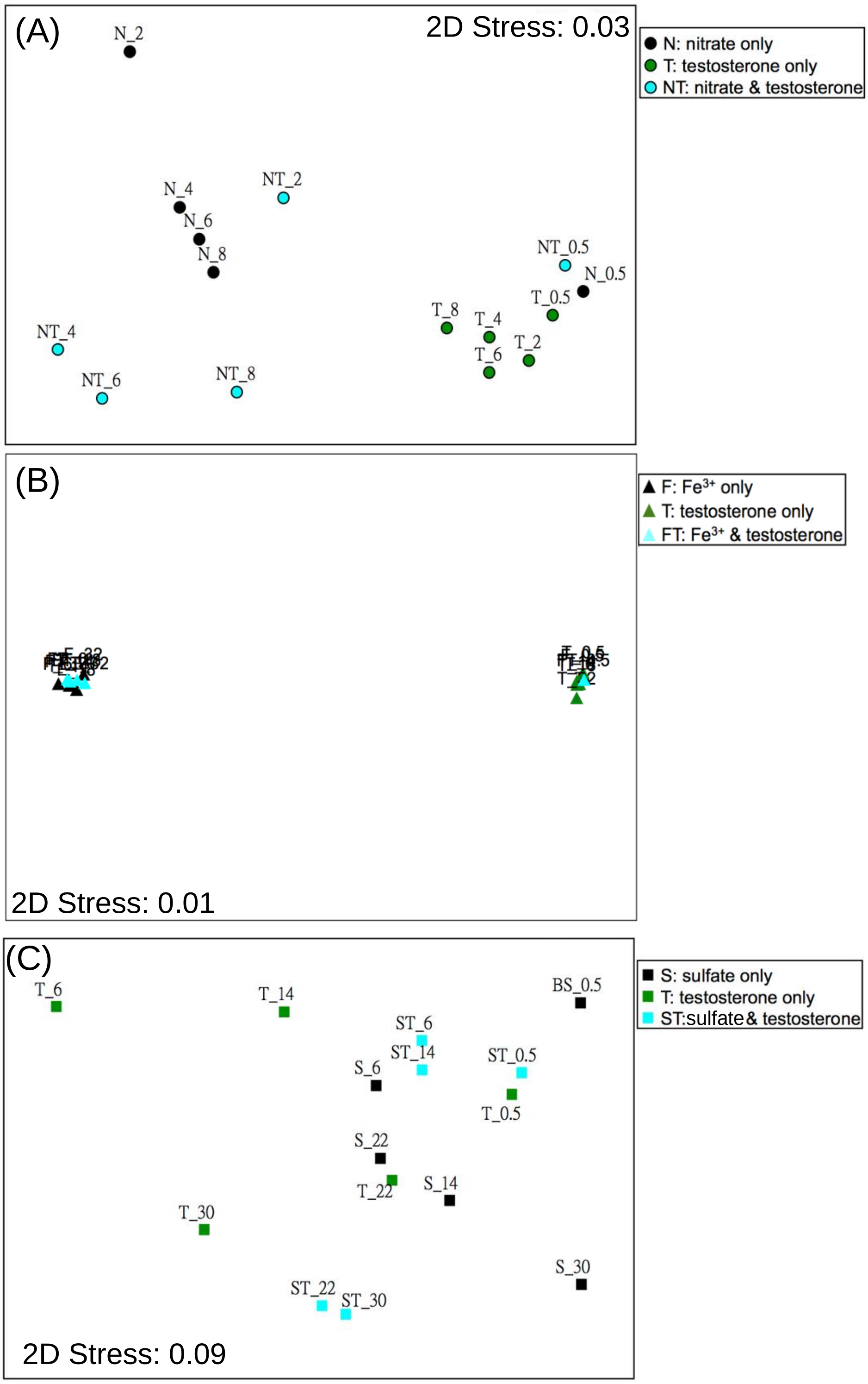

Figure S7. Non-metric multi-dimensional scaling (nMDS) plot of Bray–Curtis similarities for temporal change of bacterial community structures in the Guandu sediment treatments. (A) The subsurface layer sediment–river water mixtures spiked with nitrate or testosterone or both. (B) The middle layer sediment–river water mixtures spiked with Fe^{3+} or testosterone or both. (C) The bottom layer sediment–river water mixtures spiked with sulfate or testosterone or both. Numbers in the plot represent incubation days. Significant difference was observed among community structures in the subsurface layer (ANOSIM global $R = 0.397$, $p = 0.016$, permutation = 999, n = 15) and middle layer sediment treatments ($R = 0.395$, $p = 0.019$, permutation = 999, n = 15). No significant difference was observed among bacterial communities in the bottom layer sediments with different treatments ($R = 0.22$, $p = 0.371$, permutation = 999, n = 15).
