# Supplementary material for: Biochemical Mechanisms and Microorganisms Involved in Anaerobic Testosterone Metabolism in Estuarine Sediments
Source: Front Microbiol. 2017 Aug 11;8:1520. doi: 10.3389/fmicb.2017.01520 (PMC5554518; doi:10.3389/fmicb.2017.01520)
Supplement: Supplementary file 6 [file Image_1.PDF]

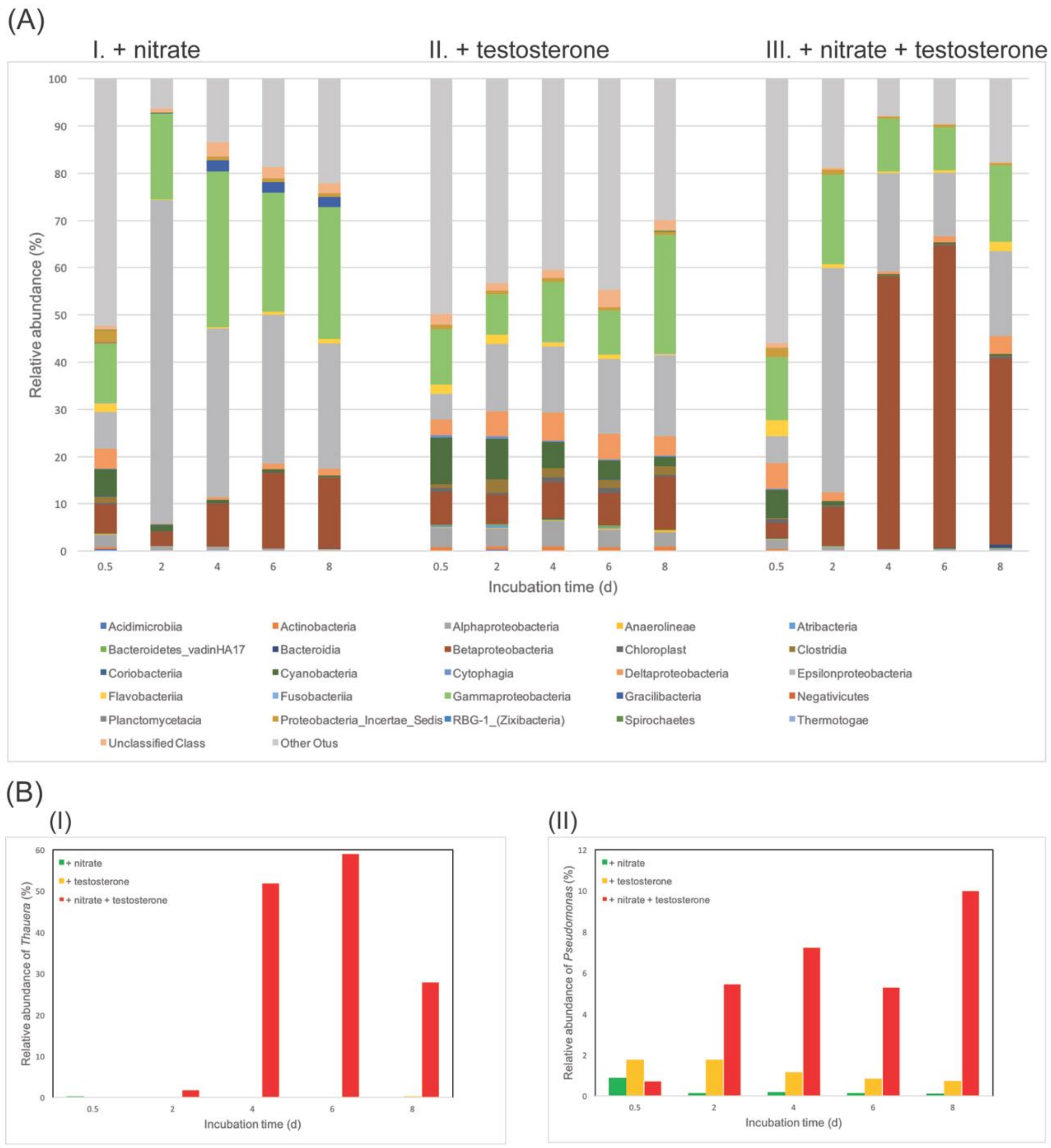

Figure S8. Temporal changes of the bacterial community structures in subsurface layer sediment (0-5cm depth)-river water mixture spiked with testosterone or nitrate or both. (A) Class-level phylogenetic analysis (Illumina MiSeq). (B) *Thauera* spp. (I) and *Pseudomonas* spp. (II) were enriched in subsurface layer sediment-river water mixture spiked with testosterone and nitrate. Individual Otus with the relative percentage below 1 % are grouped as "Other OTUs". Reads derived from the subsurface layer sediment treatments were re-analyzed using USEARCH and Mothur. The assemblage of pair-end reads, primer removal, quality filtering, chimera and singleton detections were implemented using the sequence analysis tool, USEARCH (version 10.0.240). The OTUs distribution is shown in Table S3. The assignment of OTUs to taxonomy (Table S4) was performed against the Silva database (version 128) by using Mothur (version 1.35.1).
